# Supplementary material for: Bank-firm relationships and corporate ESG greenwashing
Source: PLoS One. 2025 Jun 17;20(6):e0324596. doi: 10.1371/journal.pone.0324596 (PMC12173423; doi:10.1371/journal.pone.0324596)
Supplement: Attachment — Submitted filename: PONE-D-25-09658 comments.docx [file pone.0324596.s002.docx]

Referee Report for Manuscript: "Bank-Firm Relationships and Corporate ESG Greenwashing"

Summary: This manuscript investigates how bank-firm relationships, specifically through bank equity holdings and executive affiliations with banks, affect corporate greenwashing behaviors in Chinese A-share listed firms. The study utilizes Institutional Theory, Agency Theory, and Legitimacy Theory to explain motivations behind symbolic ESG disclosures, focusing on executive compensation and financialization as underlying mechanisms. I hope my comments can be useful to further improve this draft.

1. **Conceptual Clarity**: The paper could benefit from additional clarity on why the differences between ESG scores from various sources accurately measure greenwashing. More justification of the validity and reliability of these measures would strengthen the analysis.
2. **Endogeneity Concerns**: Although the study uses fixed effects to address endogeneity, additional econometric strategies such as instrumental variable approaches or quasi-experimental designs would strengthen causal inference. At minimum, incorporate a lead-lag analysis to address possible reverse causality and strengthen the robustness of causal inferences.
3. **Methodological Robustness**: Provide stronger rationalization for employing Tobit models and Propensity Score Matching. What are the treatment groups and control groups? The current draft is not clear on this.
4. **Empirical Analysis**:
   a. Given existing literature highlighting banks' influence through lending relationships, explicitly control for bank lending relationships to isolate the distinct impacts of equity holdings and executive affiliations on greenwashing behaviors. This additional control will strengthen causal claims and clarify contributions beyond traditional lending relationships.
   b. The current construction of the main independent variable (BankRel) combines distinct dimensions—firm holdings of bank shares, bank shareholding in the firm, and executives' banking backgrounds. Separately analyze these components, particularly distinguishing the effects of firm holding bank shares from banks holding firm shares, as these relationships may differently influence corporate governance and ESG reporting incentives. As shareholders, banks should have enhanced monitoring capabilities over firms' ESG disclosures due to their closer involvement in corporate governance, thereby potentially reducing information asymmetry. Explore further heterogeneity, potentially considering the separate effects of bank shareholding versus executive banking experience.
5. **Minor**: There are some typos and grammatical issues (e.g., consistently correct "Banking-frim" to "Banking-firm"). Standardize variable naming throughout the manuscript to improve readability, such as “Greenwashing” vs. “Green washing”.
